# Supplementary material for: Chromothripsis during telomere crisis is independent of NHEJ, and consistent with a replicative origin
Source: Genome Res. 2019 May;29(5):737–49. doi: 10.1101/gr.240705.118 (PMC6499312; doi:10.1101/gr.240705.118)
Supplement: Supplemental Material [file supp_gr.240705.118_Supplemental_file_1.zip › contigs/annotated_contigs/DB51/contig.3.DB51_length_666_mean_cov_5.36336336336.docx]

**DB51_length_666_mean_cov_5.36336336336**

```
ATCAATATTTTTGTCCTCACATCTGACTATGTAAGGAAGTTTCACAGCAAGAGTTCTATAGTGTTCTTGACAGACGTGGCAATAACAAT
 >chr18:66748962-66749205 - E=2e-134
GTTAAGATAATATTGCAAAAGTAAAGAGCTGCATGAATATTCTAATCACAGGTTTCTTAAGCAGTGACTCCTGTGTGCCTATGGATTAC

ATAAACTAAGAAATATAAACCTCTGTCATACAAATGACTACGTTTAATGTTAAATCATGAAAATA|A|CATTCTGTGTTTTTAAAATTT
                                                                   >chr18:68501556-6850
TCCTTAAGACTTTCTTTTTGATCCAAAGGTTATTTACAAGCGTTTCATTTTGTTTGCCATTACGTGATGATTTTTCTTGTCATCGATCT
1724 + E=3e-71
CTTATTGAGTTCTACTTTTATTCTATTTTGGTAAAGAAATGCTGTGTCTGATTTTAG|T|GCAATTAACATTTTTTACATACTCTGATT
                                                          >chr18:68644602-68644832 - E=
ACTGAATTTTAAGGGAGGTAAGATCTTCAACTTTTCTAGATATTGTCATTTATTCTTGCAGAGTATGGACTTTATTTCATCATCAGTGT
1e-126
TTGAGTTGTTTTTCTTCCTTATAATTACCAACATGATACTGAGTCAAATGGTATTAAGTTGATGCCTTAATGTACATAAACCTACTTAC

TAGTGATGTTAAGCACTTTTTT|TTTTTTTTTTTTCTCTCTTTATCTC
```
